# 2B4 co-stimulation and dasatinib modulation enhance anti-CD19 CAR-NK-92 cell cytotoxicity

**DOI:** 10.3389/fimmu.2025.1675877

**Published:** 2025-12-12

**Authors:** Matheus Henrique dos Santos, Júlia Teixeira Cottas de Azevedo, Mara Elisama da Silva Januário, Dayane de Fátima Schmidt, Mariane Cariati Tirapelle, Alison Felipe Bordini Biggi, Sima Ebrahimabadi, Renata Nacasaki Silvestre, Dimas Tadeu Covas, Rodrigo T. Calado, Virgínia Picanço-Castro

**Affiliations:** 1 Center for Cell-based Therapy (CTC), Regional Blood Center of Ribeirão Preto, University of São Paulo, Ribeirão Preto, São Paulo, Brazil; 2 Department of Hemotherapy and Cellular Therapy, Hospital Israelita Albert Einstein, São Paulo, Brazil; 3 School of Ribeirão Preto, University of São Paulo, Ribeirão Preto, São Paulo, Brazil

**Keywords:** CAR-NK-92 cells, NK-92, adoptive cell therapy, 2B4, DAP12, B-cell lymphoma, allogeneic therapy, dasatinib

## Abstract

**Introduction:**

Chimeric Antigen Receptor (CAR)–based therapies have transformed cancer treatment, especially in hematological malignancies. While the impact of co-stimulatory domains on CAR-T cell efficacy is well established, the optimal signaling modules for CAR-natural killer (CAR-NK) cells remain less defined. Identifying NK-tailored co-stimulatory domains is essential for maximizing CAR-NK cytotoxicity and clinical potential.

**Methods:**

Using the NK-92 cell line as a controlled proof-of-concept platform, we engineered CAR19 constructs incorporating NK-specific co-stimulatory domains, including 2B4 and DAP12. We performed functional assays to quantify cytotoxicity and cytokine production, and conducted transcriptomic profiling to evaluate transcriptional programs associated with each CAR design. To assess pharmacologic modulation, we exposed CAR-NK cells to transient dasatinib treatment and evaluated its reversible effects on CAR signaling and function. In vivo antitumor activity was tested in a xenograft model.

**Results:**

Both 2B4- and 2B4-DAP12–containing CARs enhanced NK cytotoxic programming as demonstrated by functional assays and transcriptomic signatures. Short-term dasatinib exposure reversibly suppressed CAR-NK effector function but led to enhanced activity upon drug withdrawal. In vivo, 2B4-DAP12 CAR19-NK-92 cells pretreated with dasatinib displayed superior tumor control relative to conventional 4-1BBζ CAR19-NK-92 cells.

**Discussion:**

These results highlight the importance of selecting NK-specific co-stimulatory domains and leveraging reversible Src-family kinase inhibition to optimize CAR-NK performance. The use of NK-92 cells enabled controlled mechanistic dissection of CAR signaling and pharmacologic effects, providing insights with translational relevance for engineering next-generation CAR-NK therapies in primary NK cells.

Chimeric Antigen Receptor (CAR)-based therapies have transformed cancer treatment, especially for hematological malignancies. While the choice of co-stimulatory domains is a well-established determinant of CAR-T success, the optimal signaling modules for CAR-natural killer (CAR-NK) cells remain less defined. In this proof-of-concept study, we used the NK-92 cell line as a controlled experimental platform to evaluate CAR constructs incorporating NK-specific co-stimulatory domains, including 2B4 (CD244) and DAP12. Functional assays and transcriptomic profiling demonstrated that 2B4- and 2B4-DAP12–based CARs promoted NK cytotoxic programming. We further explored transient pharmacologic modulation with dasatinib, showing that short-term exposure reversibly suppressed CAR-NK activity but enhanced function upon withdrawal. *In vivo*, 2B4-DAP12 CAR19-NK-92 cells pretreated with dasatinib achieved superior tumor control compared to conventional 4-1BBζ CAR19-NK-92 cells. These findings underscore the value of different settings of co-stimulatory domains and reversible kinase inhibition as strategies to optimize CAR design. Importantly, by employing NK-92 cells as a proof-of-concept system, this work provides mechanistic insights that will guide the development of next-generation CAR-NK therapies in primary NK cells.

## 1 Introduction

Cell-based immunotherapies have significantly advanced cancer treatment, particularly in hematologic malignancies resistant to standard therapies. Chimeric antigen receptors (CAR) expressed on T cells or CAR-T therapies targeting CD19 have demonstrated unprecedented clinical success, resulting in the FDA approval of multiple CAR-T products (1). However, limitations including manufacturing complexity, high costs, and patient-specific challenges such as T-cell lymphopenia remain (2, 3). Natural killer (NK) cells represent a promising allogeneic alternative due to their innate cytotoxicity and lack of HLA restriction (4). This allows for the development of allogeneic NK cell-based therapies, overcoming the need for patient-specific cell collection. NK cell function is tightly regulated by a balance between activating and inhibitory receptors (5, 6), which dictates their cytolytic capacity and cytokine/chemokine secretion (7). NK cell-based adoptive therapies have shown promising results in clinical trials, demonstrating their safety and low toxicity (8, 9). However, while NK cells derived from peripheral blood (PB-NK) or umbilical cord blood (CB-NK) have demonstrated efficacy against hematologic cancers, their activity against solid tumors remains limited (10). Despite encouraging safety profiles and early clinical responses, CAR-NK cells have yet to reach the efficacy of CAR-T cells. A possible barrier is the lack of NK-specific optimization in current CAR constructs, which often utilize T cell-derived signaling domains. Current clinical trials and research primarily utilize PB-NK, CB-NK, or NK-92 cells genetically engineering with CAR constructs to explore and address these challenges and improve their anti-tumor activity (11–15).

Recent evidence suggests that incorporating NK-specific co-stimulatory domains, such as 2B4 (CD244) and DAP12, may enhance CAR-NK functionality by engaging NK cell-optimized activation pathways (16). Multiple studies have demonstrated that integrating the 2B4 intracellular domain as a co-stimulatory module in CAR constructs enhances CAR-NK cell cytotoxicity in both *in vitro* and *in vivo* models (16–18). However, the exact signaling mechanisms activated by 2B4 remain unclear. Another promising signaling molecule is DAP12, a transmembrane adaptor protein involved in NK cell activation through receptors such as NKG2C, NKp44, and KIRs (19). CAR constructs incorporating DAP12 as a co-stimulatory domain have demonstrated superior activation potential compared with conventional CD3ζ-containing CARs (20). Most CAR constructs rely on CD3ζ to trigger cellular activation (21). The CAR-mediated signaling cascade involves the phosphorylation of SRC-family kinases such as LCK, which subsequently phosphorylates CD3ζ and ZAP70. This cascade ultimately leads to the activation of transcription factors, including Nuclear factor of activated T-cells (NFAT) and Nuclear Factor kappa-light-chain-enhancer of activated B cells NF-κB, which regulate cellular functions (22, 23). Given the unique signaling networks of NK cells, we hypothesized that substituting the conventional 4-1BB and CD3ζ domains with NK-associated activation modules, such as 2B4 and DAP12, could improve CAR NK cell functionality.

To further optimize CAR-NK cell performance, we investigated dasatinib, a tyrosine kinase inhibitor (TKI), as a potential pharmacological modulator. Originally developed to inhibit BCR-ABL (24), dasatinib has been approved as the first-line treatment for Philadelphia chromosome-positive chronic myeloid leukemia and acute lymphoblastic leukemia. Additionally, transient pharmacological modulation using tyrosine kinase inhibitors like dasatinib has been shown to suppress CAR-T cell exhaustion and may similarly benefit CAR-NK cells (25, 26).

Here, we evaluated the impact of 2B4-DAP12 co-stimulation and transient dasatinib treatment on CAR-NK-92 cells targeting CD19+ B-cell malignancies. Our findings revealed that dasatinib-mediated suppression of CAR-NK cell activity is fully reversible and non-toxic, with post-withdrawal recovery leading to enhanced cytotoxic function. Furthermore, RNA sequencing data revealed that CAR-NK cells incorporating 2B4 signaling exhibited enrichment in cytotoxicity pathways, degranulation markers, calcium flux regulators, and pro-inflammatory cytokine networks. *In vivo*, CAR19-2B4ζ and CAR19-2B4DAP12 NK-92 cells successfully delayed tumor progression and improved survival compared to the 4-1BBζ constructs. These effects were further enhanced by transient dasatinib pre-conditioning. Taken together, these results support a combined strategy of NK-optimized costimulation and pharmacological modulation with dasatinib to improve CAR-NK therapeutic efficacy.

## 2 Materials and methods

### 2.1 Cells and culture conditions

Natural Killer (NK-92) cell line (ATCC, CRL-2407™) was cultured in serum-free X-VIVO 10 medium (Lonza, Cologne, Germany) supplemented with 5% heat-inactivated human AB plasma (sourced from the Regional Blood Center of Ribeirão Preto, Brazil) and 200 IU/mL IL-2 (Clinigen), as previously described (27). The human leukemia cell lines Nalm-6 (CRL-3273), Raji (CCL-86), K562 (ATCC, CCL-243), and Namalwa (CRL-1432) were maintained in RPMI medium (Thermo Fisher Scientific) supplemented with 10% fetal bovine serum (FBS). All cell lines tested negative for mycoplasma contamination using the MycoAlertPLUS Mycoplasma Detection Kit (Lonza) (reference value < 1.0). Authentication was performed via short tandem repeat (STR) analysis, which identifies human cell lines by comparing the major STR loci. A minimum of eight STR loci were amplified using specific primers and a PCR amplification kit (Sigma-Aldrich), targeting loci D5S818, D7S820, CSF1PO, vWA, D16S539, TPOX, THO1, and D13S317. Polyacrylamide gel electrophoresis followed by silver nitrate staining was used for the analysis. STR Profiles: NK-92: CSF1PO: 11,12; D13S317: 9,12; D16S539: 11,12; D5S818: 12,13; D7S820: 10,11; THO1: 6,9.3; TPOX: 8; vWA: 18. Raji: CSF1PO: 10,12; D13S317: 13; D16S539: 8,11; D5S818: 10,13; D7S820: 10; THO1: 6,7; TPOX: 8,13; vWA: 16,19. Nalm-6: CSF1PO: 12; D13S317: 9,12; D16S539: 10,11; D5S818: 11,13; D7S820: 8,11; THO1: 8,9; TPOX: 8,10; vWA: 15,16. Namalwa: CSF1PO: 10,11; D13S317: 11,12; D16S539: 9; D5S818: 12,13; D7S820: 11; THO1: 7,9.3; TPOX: 6,11; vWA: 14. K562: (CSF1PO: 9,10; D13S317: 8; D16S539: 11,12; D5S818: 11,12; D7S820: 9, 11; THO: 9.3; TPOX: 8,9; vWA: 16).

### 2.2 Construction of lentiviral vectors and transduction of NK-92 cells

**Figure 1:**
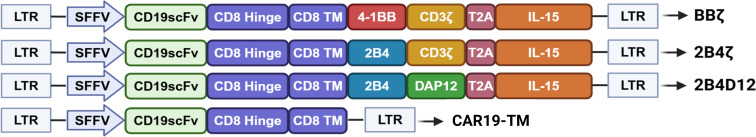
Schematic representation of CAR constructs targeting CD19 under the control of the spleen focus-forming virus (SFFV) promoter. Each CAR includes a CD19 scFv, a CD8 hinge and transmembrane domains, co-stimulatory domains, a self-cleaving T2A peptide, and a sequence of soluble cytokine IL-15.

Lentiviral vectors encoding CAR19-IL-15 were designed and constructed as previously described (27) with transgene expression driven by the Spleen Focus-Forming Virus (SFFV) promoter. The CAR constructs consisted of a CD19 scFv, CD8α hinge, and CD8 transmembrane domain. Cytoplasmic signaling domains, including CD137 (4-1BB), 2B4, DAP12, and CD3ζ, were used to generate NK-specific CARs (**Figure 1**). A vector containing the CD19 scFv, CD8 hinge, and transmembrane domains were used as a signaling control. The CAR construct sequences were validated by restriction enzyme digestion and sequencing analyses. Lentiviral production was performed by transient co-transfection of HEK293T cells using a third-generation lentiviral packaging system as previously described (28). Lentiviral vector titers were determined by transducing K562 cells, followed by flow cytometric analysis to assess the transduction efficiency. NK-92 cells were transduced with lentiviral particles at a multiplicity of infection (MOI) of 10.

### 2.3 Magnetic enrichment of CAR-NK cells

Between 7 and 14 days post-transduction, NK-92 cells expressing CARs underwent positive selection using magnetic beads. A total number of 1×10^6^ to 10×10^6^ cells were incubated with Biotin-SP-conjugated AffiniPure F(ab’)_2_ Fragment Goat Anti-Mouse antibody (200 µL PBS, 40 minutes on ice). The cells were washed, resuspended in PBS, and incubated with anti-biotin magnetic beads (Miltenyi Biotec) for 15 minutes at room temperature. The cell suspension was applied to MS columns (Miltenyi Biotec) positioned in a magnetic stand. Unbound cells were discarded, whereas CAR-positive cells retained in the column were eluted and resuspended in fresh medium.

### 2.4 *In vitro* cytotoxicity assay by flow cytometry

Raji (CD19^+^), NALM-6 (CD19^+^), and Namalwa (CD19^+^) cells were labeled with PKH67 using a Green Fluorescent Cell Linker Kit for General Cell Membrane Labeling (PKH67GL, Merck). For assays in which dasatinib treatment was considered, effector cells were cultivated for 24 hours with determined concentrations of dasatinib (Sigma-Aldrich). After incubation, the cells were washed twice with PBS and resuspended in X-VIVO medium for the respective assays. As treatment control, effector cells were cultivated in DMSO. Effector and target cells were co-cultured in 96-well plates at various effector-to-target (E:T) ratios. Cytotoxicity was assessed by flow cytometry, measuring the reduction in viable PKH67-positive target cells relative to conditions containing only target cells, using the following formula: % target lysis = 100 × (%remaining viable target cells alone - %remaining viable target cells against effector cells)/%remaining viable target cells alone.

### 2.5 Quantification of IL-15, IFN-γ, and TNF-α

IL-15 levels in the cell culture supernatant were measured using a Human IL-15 ELISA kit (Invitrogen Catalog BMS2106). IFN-γ and TNF-α concentrations were quantified by ELISA, following the manufacturer’s protocol (BD Biosciences).

### 2.6 Assessment of NK cell degranulation via CD107a expression

NK cell degranulation was evaluated by measuring CD107a (LAMP-1) expression during activation. A total of 3×10^5^ NK cells per well were cultured in 96-well plates for 5 hours, either alone (control) or co-cultured with Raji, Nalm-6, Namawa or K562 target cells (3×10^5^ cells per well) at an effector-to-target (E:T) ratio of 1:1. To monitor degranulation, NK cells were stained with anti-CD107a-PE antibody (BD Biosciences) or an isotype as a control. After one hour of incubation, 2 µM monensin (Sigma-Aldrich) was added to inhibit vesicular trafficking. CD107a expression was analyzed using flow cytometry, gating the CD56^+^ NK cell population to assess degranulation activity.

### 2.7 Bulk RNA sequencing

RNA was extracted from magnetically selected CAR-NK cells after 5 hours of cytotoxicity assay against Nalm-6 cells at a 1:1 ratio for each sample. Total RNA from all CAR-NK cells was extracted using the RNeasy Plus Micro kit (Qiagen, Hilden, Germany) and quantified using a Nanovue spectrophotometer (GE Healthcare, Chicago, Illinois, USA). Poly(A) RNA sequencing libraries were generated using Illumina’s TruSeq Stranded mRNA protocol and were processed by LC Sciences (Houston, TX, USA).

To isolate polyadenylated mRNAs, oligo-dT magnetic beads were used for two rounds of purification. The mRNA was then fragmented into a divalent cation buffer at an elevated temperature. Quality control and quantification of the sequencing libraries were performed using an Agilent 2100 Bioanalyzer equipped with a high-sensitivity DNA chip. Paired-end sequencing was performed using the Illumina NovaSeq 6000 platform.

Differential expression analysis was performed using StringTie and transcript abundance was measured in fragments per kilobase of transcript per million reads (FPKM). Differentially expressed mRNAs in CAR-NK cells were identified using edgeR, applying a log_2_ fold change threshold of >1.5 or <−1.5, with statistical significance set at *p* < 0.05. Heatmap and GSEA were performed using the PyDeseq2 package in Python. RNA-seq data and supporting datasets are available at the NCBI Gene Expression Omnibus (GEO) and are accessible through GEO Series accession number GSE306760.

### 2.8 Establishment and monitoring of a B-cell lymphoma xenograft model

Immunodeficient NSG mice (NOD.Cg-Prkdcscid Il2rgtm1Wjl/SzJ) aged 10–14 weeks were assigned to different experimental groups, including NK-92 and CAR19-NK-92 cells (CAR19-BBζ, CAR19-2B4ζ, or CAR19-2B4D12). Tumor engraftment was established by injecting 1×10^4^ Nalm6-Luc^+^ cells previously transduced for D-luciferase expression into the caudal lateral vein.

Effector cells (5×10^6^) were administered via the same route on days 1, 4, 8, 12, and 17 post-tumor inoculation. Tumor progression was tracked by bioluminescence imaging using the IVIS Lumina System (PerkinElmer, Waltham, MA, USA). Bioluminescence signals were recorded 5–10 minutes after intraperitoneal administration of 150 mg/kg d-luciferin (PerkinElmer). All applicable guidelines for animal care and use were followed and animal experiments were approved by the responsible government committee in Brazil (n. 012/2021).

### 2.9 Statistics

Comparisons between two groups with a normal distribution were conducted using Student’s t-test, and for more than two groups, one-way ANOVA followed by Tukey’s *post-hoc* test was employed. In cases of non-normal distribution, the Mann-Whitney U test was used to compare two groups, and the Kruskal-Wallis test with Dunn’s *post-hoc* test was used to compare more than two groups. * P ≤ 0.05, ** P ≤ 0.01, *** P ≤ 0.001, and **** P ≤ 0.0001. Statistical analyses were performed using GraphPad Prism software version 9.

### 2.10 Ethics statement

All animal procedures were approved by the Institutional Animal Care and Use Committee (CEUA) of the University of São Paulo (Protocol 012/2021) and conducted in accordance with ethical guidelines. Ethical approval was obtained from Brazilian National Research Ethics Commission (CEP/CONEP, #7.485.958); and, for CAR-NK–related activities, the National Biosafety Technical Commission (CTNBio, #SEI 01245.007960/20224-76), Brazil. All participants provided written informed consent prior to inclusion in the study. The study was conducted in accordance with the local legislation and institutional requirements.

## 3 Results

### 3.1 Enriched population of transduced NK-92 cells maintains stable CAR19 expression over time across different CAR constructs

Our study screened two CAR constructs optimized for NK cell activity. All CARs targeted CD19 using anti-CD19 scFv (HD37 clone), CD8 hinge, and transmembrane domain. As co-stimulatory domains, we tested 2B4 and/or DAP12 and compared them to the conventional CAR construct containing 4-1BB and CD3ζ, as well as with a control construct lacking the intracellular signaling domains, CAR19-TM.

**Figure 2:**
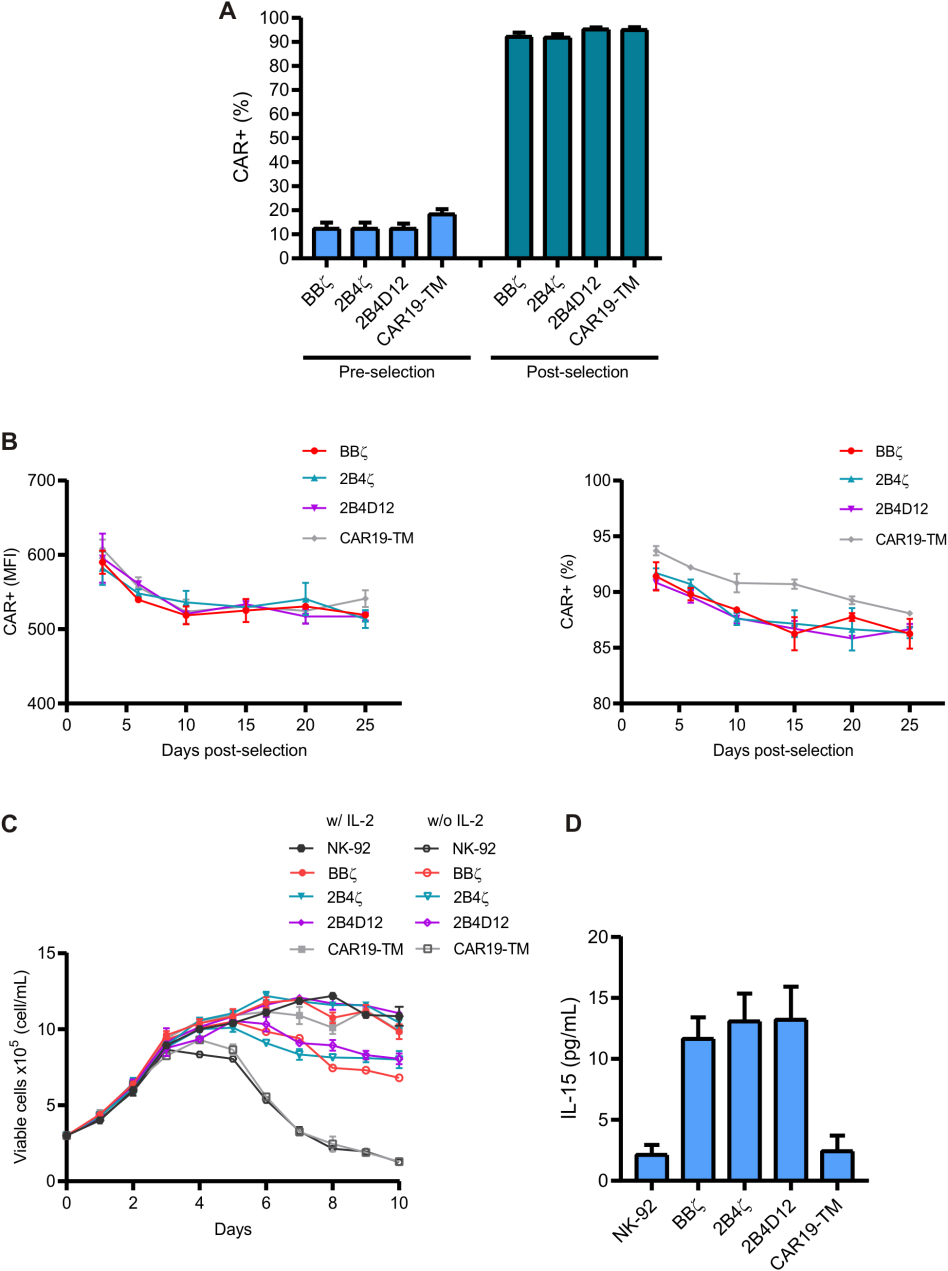
Selection and characterization of CAR19-NK-92 cells. **(A)** NK-92 cells were transduced with the CAR constructs with an MOI of 10. CAR expression was assessed three days after transduction and after immunomagnetic enrichment. **(B)** The percentage and mean fluorescence intensity (MFI) of CAR expression were assessed over 25 days post-selection. **(C)** The growth kinetics of viable cells cultured in medium with or without 200U/mL of IL-2 were monitored over 10 days. **(D)** The IL-15 concentration in the culture supernatant was measured by ELISA after 48 hours of culture.

To efficiently evaluate the ability of CARs, NK-92 cells were transduced with lentiviral particles at a multiplicity of infection (MOI) of 10, carrying the sequences for the fourth-generation anti-CD19 CAR constructs (**Figure 1**). Transduction efficiency was consistent across all constructs, with 10–20% of cells expressing CAR three days post-transduction (**Figure 2A**). CAR19-NK-92 cells were enriched by immunomagnetic positive selection, resulting in a population of over 90% CAR-positive cells (**Figure 2A**).

After enrichment, CAR19-NK-92 cells were cultured for 25 days to assess the stability of CAR expression. A slight decrease in fluorescence intensity and percentage of CAR-positive cells was observed within the first ten days after post-selection. However, CAR expression remained stable throughout the 25-day culture period (**Figure 2B**). This demonstrated that the enrichment process effectively generated a consistent CAR-expressing cell population across all constructs.

To assess cell proliferation and viability, non-transduced NK-92, CAR19-NK-92, and control CAR19-TM cells were cultured with or without IL-2, which is essential for NK-92 cell growth. CAR-NK-92 cells, which secrete IL-15, exhibited prolonged viability in culture compared to non-transduced NK-92 and CAR19-TM cells (**Figure 2C**). Moreover, increased levels of IL-15 were detected in the culture supernatants of CAR-NK-92 cells, indicating that IL-15 secretion played a role in supporting their extended survival (**Figure 2D**).

### 3.2 CAR19 incorporating 2B4 co-stimulatory domain and CD3ζ or DAP12 demonstrates comparable cytotoxicity to CAR19-41BB-CD3ζ *in vitro*

To further evaluate the specific cytotoxicity of CAR19-NK-92 cells, we tested their activity against CD19-positive B cell lines, including Nalm-6 (derived from a patient with acute lymphoblastic leukemia), Raji, and Namalwa (both derived from patients with Burkitt lymphoma) as well as the CD19-negative cell line K562 (derived from a patient with chronic myelogenous leukemia) as negative control.

**Figure 3:**
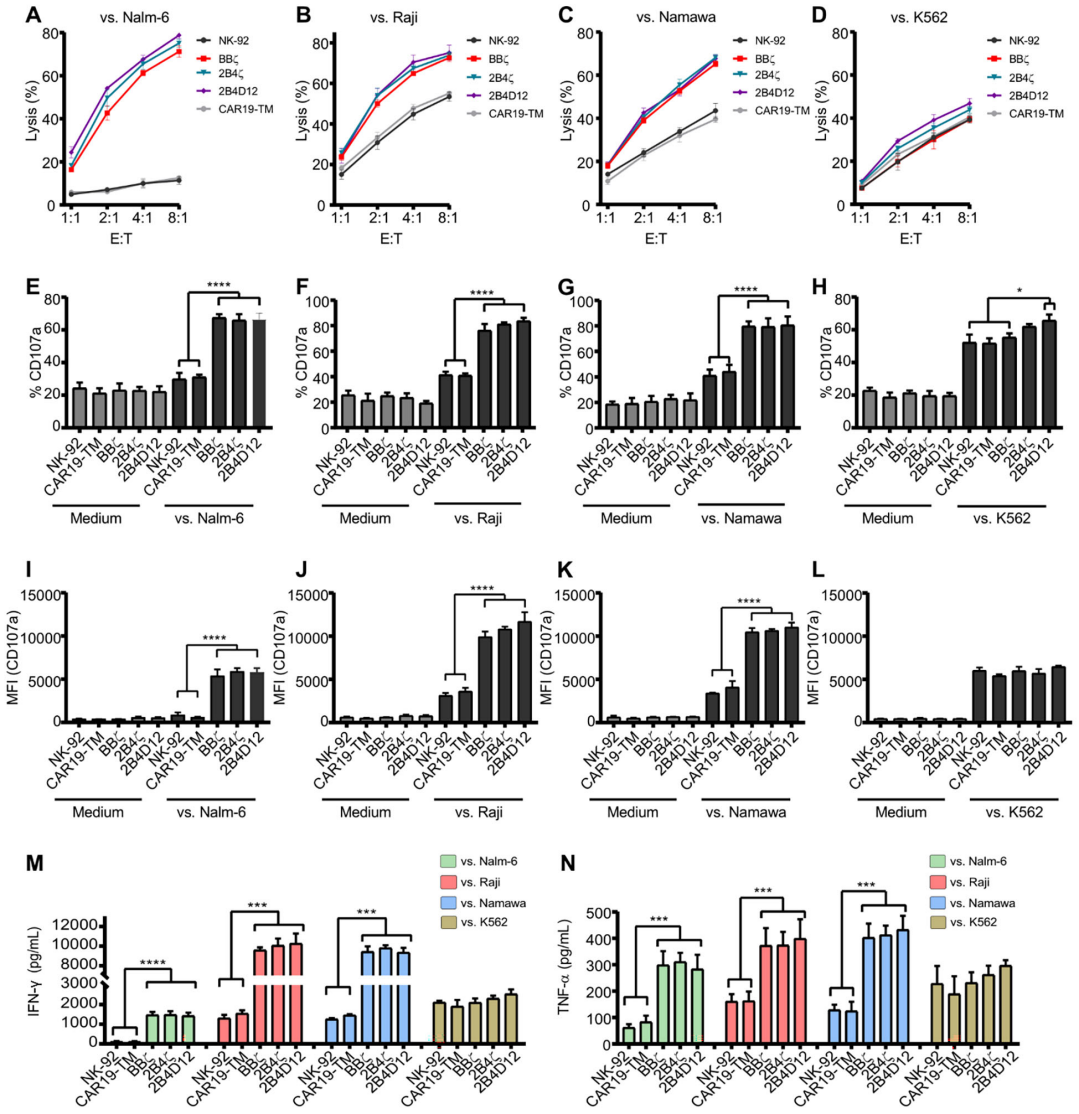
Functional characterization of CAR19-NK-92 and NK-92 cells against target cells. Co-culture assays were performed with CD19-positive cell lines (Nalm-6, Raji, and Namalwa) and CD19-negative cell line K562. **(A-D)** Target cells were labeled with PKH67 and incubated at different effector-to-target (E:T) ratios with non-transduced NK-92 cells or CAR19-NK-92 cells for 5 hours. Target cells’ viability was assessed by flow cytometry. **(E-L)** Degranulation assays were conducted by measuring CD107a expression, represented as percentage **(E-H)** and the mean fluorescence intensity (MFI) **(I-L)**, after co-culture with PKH67-labeled target cells for 5 hours at a 1:1 ratio with non-transduced NK-92 or CAR19-NK-92 cells. **(M, N)** Quantification of pro-inflammatory cytokine production in the culture supernatant after 5 hours of co-culture between non-transduced NK-92 or CAR19-NK-92 cells and target cells at a 1:1 ratio. Cytokine levels (IFN-γ and TNF-α) were measured using ELISA. Values are expressed as mean ± SD. *p < 0.05, **p < 0.01, ***p < 0.001, ****p < 0.0001, according to one-way ANOVA. *n* = 6.

In co-culture assays, all CAR19-NK-92 cells demonstrated significantly enhanced cytotoxicity against Nalm-6, Raji, and Namalwa cells compared to unmodified NK-92 cells (**Figures 3A-C**). Specifically, when co-cultured with CD19-positive target cells, all CAR constructs exhibited a marked increase in cytotoxic capacity across all four effector-to-target (E:T) cell ratios tested, outperforming non-transduced NK-92 cells or those expressing control CAR19-TM (**Figures 3A-C**). In contrast, cytotoxicity against CD19-negative K562 cells remained comparable across groups, confirming the antigen-specific activity of the CAR constructs (**Figure 3D**).

The cytotoxic activity of NK cells is closely associated with degranulation, a process characterized by the release of cytotoxic proteins, such as granzyme and perforin, which can be detected by CD107a expression. Consistent with the observed target cell death, CAR19-NK-92 cells exhibited significantly higher CD107a levels when co-cultured with CD19-positive cell lines than non-transduced NK-92 cells or those expressing the CAR19-TM construct (**Figures 3E-G**). When co-cultured against K562 cells, the expression levels of CD107a demonstrated to be comparable across the CAR19-NK-92 cells and non-CAR-NK-92 cells, except to the cells expressing 2B4D12, that showed a slightly elevated percentage of cells positive for CD107a (**Figure 3H**). Interestingly, CD107a mean fluorescence intensity (MFI) was lower in assays against Nalm-6 cells than against Namalwa and Raji (**Figures 3I-K**), suggesting that target cell characteristics, such as CD19 expression levels or other natural ligands to NK-92 may influence the degree of NK cell activation beyond CAR engagement, as Nalm-6 cells express lower CD19 levels than Raji and Namalwa cells (**Supplementary Figure S1**), which may account for the observed differences in activation. Besides the percentage of CD107a-positive cells were higher in 2B4D12-expressing cells, there were no differences in fluorescence intensity among CAR19-NK-92 cells and non-CAR-NK-92 cells (**Figure 3L**).

Furthermore, CAR19-NK-92 cells secreted significantly higher levels of the pro-inflammatory cytokines IFN-γ and TNF-α than non-transduced NK-92 cells or those expressing the CAR19-TM construct, underscoring their enhanced cytotoxic potential against CD19-positive targets (**Figures 3M, N**). It is noteworthy that CD107a expression and cytokine production did not differ between CAR19-NK-92 cells and non–CAR-NK-92 cells in the absence of target cells, suggesting that CAR expression does not induce tonic signaling. Notably, CAR constructs incorporating the 2B4 co-stimulatory domain in combination with either CD3ζ or DAP12 demonstrated cytotoxicity slightly higher to that of BBζ *in vitro*, highlighting the robust anti-tumor activity of these designs.

### 3.3 RNA sequencing analysis reveals distinct cytotoxic and cytokine-related gene signatures in CAR19-NK-92 cells

To better understand the molecular mechanisms driving the observed functional differences among CAR-NK constructs, we performed bulk RNA sequencing (RNA-seq) on CAR19-NK-92 cells after a 5-hour co-culture with Nalm-6 target cells. Differential gene expression analysis revealed that both 2B4ζ and 2B4D12 CAR19-NK-92 cells exhibited a distinct transcriptional profile compared to BBζ CAR19-NK-92 cells.

**Figure 4:**
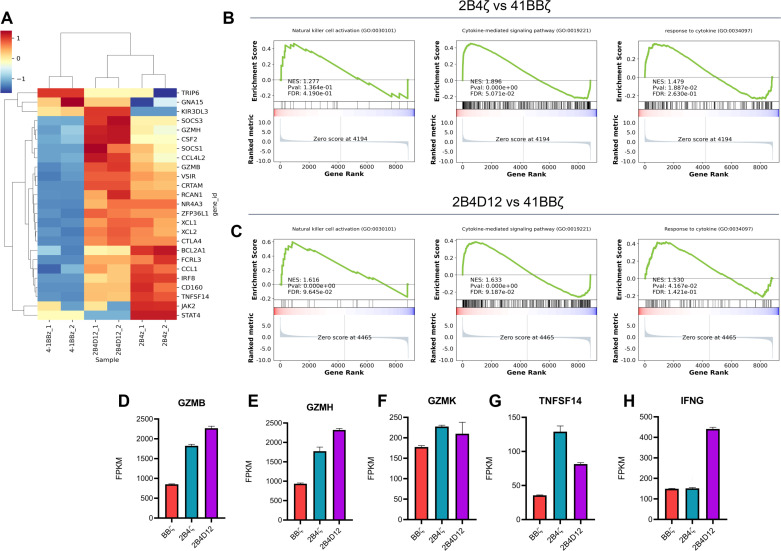
Transcriptomic profiling of CAR19-NK-92 cells incorporating different co-stimulatory domains. **(A)** Heatmap showing differentially expressed genes (DEGs) related to lymphocyte activation, cytotoxicity, cytokine signaling, and immune regulation in CAR19-NK-92 cells expressing BBz, 2B4z, and 2B4D12 after 5 hours of co-culture with Nalm-6 cells. DEGs were defined using a threshold of log2 fold change > 1 and Q < 0.05. (B, C) Gene Set Enrichment Analysis (GSEA) comparing 2B4z **(B)** and 2B4D12 **(C)** to BBz showed enrichment in pathways associated with natural killer cell activation (GO:0030101), cytokine-mediated signaling (GO:0019221), and response to cytokines (GO:0034097). **(D–F)**Bar plots displaying expression levels (FPKM) of cytotoxic effector genes GZMB, GZMH, and GZMK; **(G, H)** Expression of pro-inflammatory cytokine genes TNFSF14 and IFN-g. Values are expressed as mean ± SD.

Specifically, 25 genes were significantly upregulated (log_2_ fold change >1, Q<0.05) in both 2B4ζ and 2B4D12 CAR19-NK-92 cells compared to BBζ (**Figure 4A**). Gene Set Enrichment Analysis (GSEA) revealed significant enrichment of gene signatures associated with natural killer cell activation (GO:0030101), cytokine-mediated signaling pathways (GO:0019221), and response to cytokines (GO:0034097) in both 2B4ζ and 2B4D12 constructs compared with BBζ (**Figures 4B, C**). Notably, 2B4D12 displayed slightly stronger enrichment scores, suggesting a broader engagement of NK activation programs. Expression analysis of individual effector genes demonstrated upregulation of GZMB and GZMH, canonical cytotoxic granule components, in 2B4ζ and 2B4D12 CAR19-NK-92 cells, consistent with enhanced cytolytic potential (**Figures 4D–F**). Additionally, TNFSF14 expression was elevated in both 2B4-based constructs, and IFN-γ was particularly upregulated in 2B4D12 cells (**Figures 4G–H**), indicating a potentially greater pro-inflammatory cytokine production profile.

These transcriptomic profiles underline distinct activation states induced by different co-stimulatory domains. Despite these differences at the transcriptional level, functional assays revealed no statistically significant differences in cytokine secretion or cytotoxicity under the tested conditions. This discrepancy highlights the complexity of translating transcriptional signatures into functional outcomes and underscores the need for further studies to elucidate these findings.

### 3.4 *In vivo* cytotoxicity of 2B4ζ and 2B4D12 CAR19-NK-92 cells

**Figure 5:**
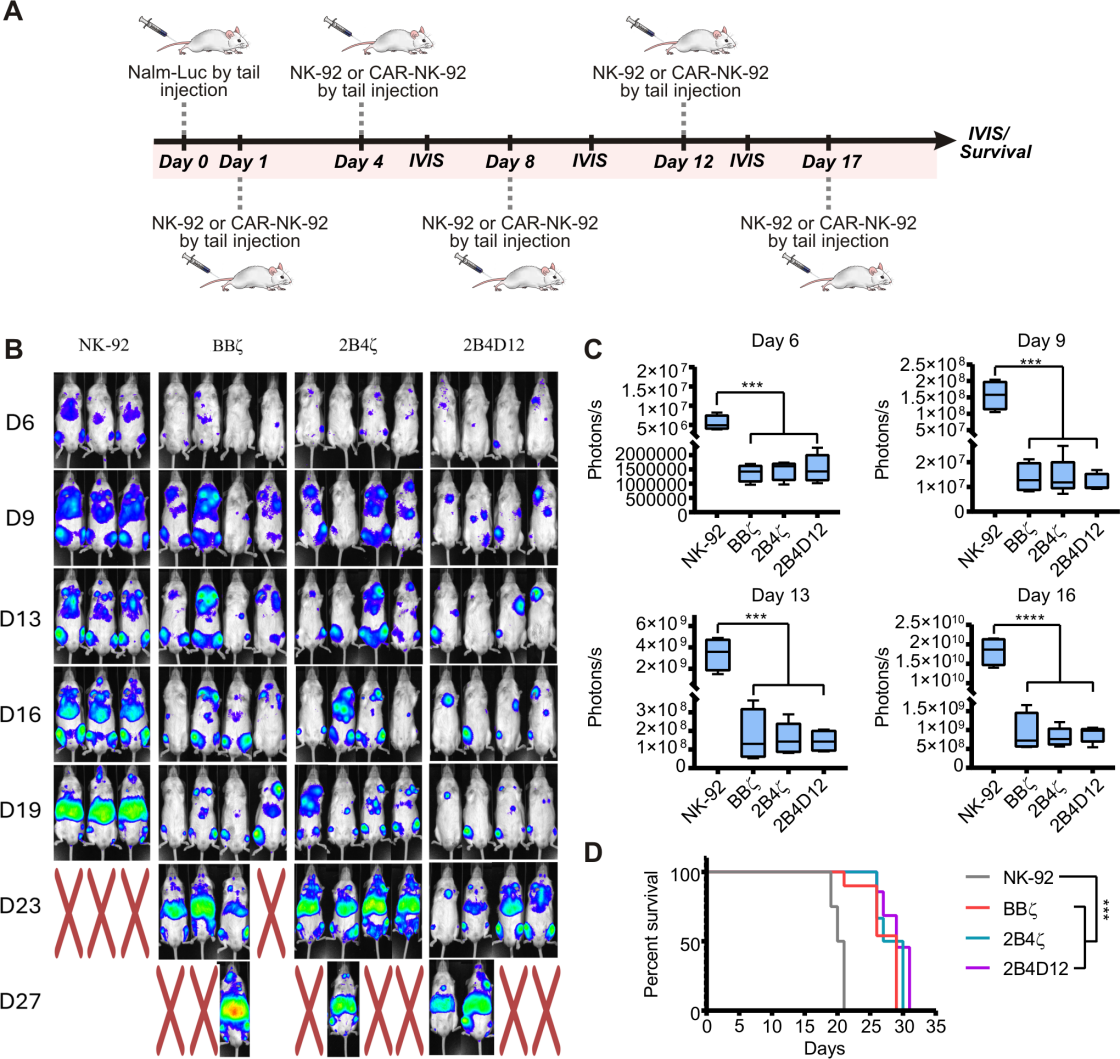
*In vivo* assessment of anti-tumor activity of CAR19-NK-92 cells. **(A)** Experimental design: Mice were intravenously inoculated with 1×10^4^ Nalm-Luciferase cells on day 0 and treated with NK-92 or CAR19-NK-92 cells (BBζ, 2B4ζ, or 2B4D12). **(B)** Tumor progression was monitored by bioluminescence, with representative bioluminescence images shown. Mice marked with “X” indicate death during the experiment. **(C)** Bioluminescence quantification showing tumor burden on days 6, 9, 13, and 16. Results are presented as mean ± SD, with significant differences determined by one-way ANOVA (****p < 0.001, ***p < 0.01, **p < 0.1). **(D)** Survival analysis of the animals represented by the Kaplan-Meier curve. Statistically significant differences were determined by the Log-rank test (***p < 0.01).

We further evaluated the antitumor activity of our new CAR19 construct using an NSG mouse model. Mice were inoculated with firefly luciferase-expressing Nalm-6 cells (Nalm-Luc) and treated with different CAR19-NK-92, cells, including BBζ, 2B4ζ, 2B4D12, and NK-92 cells as controls. Tumor burden was monitored over time using bioluminescence imaging (IVIS Lumina system), with NK cell infusions administered on days 1, 4, 8, 12, and 17 after tumor inoculation (**Figure 5A**).

The bioluminescence imaging data (**Figure 5B**) showed clear difference tumor progression and survival among the groups. Mice treated with non-transduced NK-92 cells exhibited rapid tumor progression, indicating the inability of these cells to effectively control tumor growth *in vivo*. In contrast, all CAR19-NK-92-treated groups displayed delayed tumor progression compared to the NK-92 control group, highlighting the critical role of CAR constructs in mediating CD19-specific tumor targeting and cytotoxicity (**Figure 5B**).

In the groups treated with CAR19, tumor progression was similar across the three constructs BBζ, 2B4ζ, and 2B4D12, suggesting that CARs incorporating 2B4 as a co-stimulatory domain function are comparable to those containing 4-1BB (**Figure 5C**). Additionally, these constructs were associated with prolonged survival compared with mice treated with non-transduced NK-92 cells (**Figure 5D**).

### 3.5 Dasatinib-induced reversible functional inhibition of NK-92 cells: rapid reactivation and enhanced CD19-targeted cytotoxicity with 2B4 co-stimulation

Pharmacological interventions offer a means to enhance the cytotoxicity of effector immune cells. An interesting approach involves transient inhibition of tyrosine kinases, thereby maintaining cells in a non-activated state (29). Upon withdrawal of the inhibitor, the subsequent restoration of signaling cascades may be potentiated, potentially leading to enhanced cytotoxic responses and improved functional outcomes. Dasatinib temporarily inhibits key tyrosine kinases, including SRC, LCK, and ZAP70, which are involved in downstream signaling pathways of CAR receptors (29). Short-term treatment of CAR19-NK-92 cells with dasatinib followed by its removal may restore and enhance the cytotoxic activity of CAR19-NK-92 cells against their target.

To test this hypothesis, we evaluated the effect of transient treatment with dasatinib (10, 25, 50, 100, and 200 nM) on CAR19-NK-92 cells transduced with constructs BBζ, 2B4ζ, 2B4D12, or CAR-19TM, as well as on non-transduced NK-92 cells. These cells were co-cultured with CD19-positive tumor cell lines (Nalm-6 and Raji) at an effector-to-target ratio of 2:1 (**Supplementary Figure S2A**). Dasatinib at 25 nM exhibited the most significant increase in cytotoxicity, whereas higher concentrations (100 and 200 nM) reduced the reactivation of cytotoxic activity (**Supplementary Figure S2B, C**).

**Figure 6:**
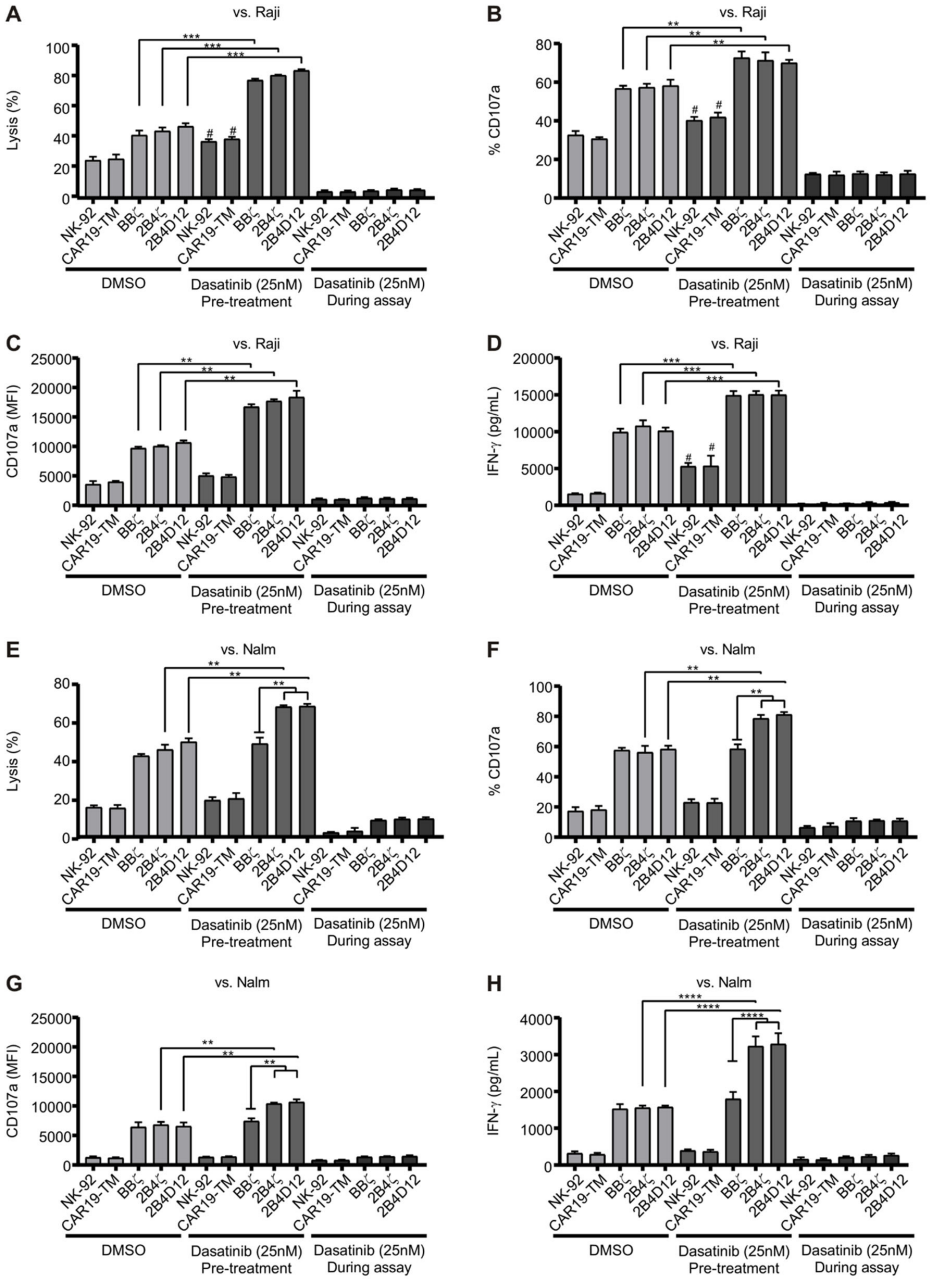
Enhanced Functionality of CAR19-NK-92 Cells Following Dasatinib Treatment. CAR19-NK-92 cells were treated with 25 nM dasatinib or DMSO for 24 hours, washed for dasatinib withdraw or maintained under treatment, and co-cultured with CD19-positive tumor cell lines (Raji or Nalm-6) at a 2:1 effector-to-target (E:T) ratio for 5 hours. The impact of dasatinib was assessed by measuring: **(A, E)** cytotoxicity via target cell viability, **(B, C, F, G)** degranulation through CD107a expression accessed by flow cytometry and **(D, H)** IFN-γ production in the culture supernatant (ELISA). Results are presented as mean ± SD. ****p < 0.001, ***p < 0.01, **p < 0.1 determined by one-way ANOVA. # represents significance of p < 0.05 of NK-92 or CAR19-TM pre-treated with dasatinib against pre-treated with DMSO. *n =* 6.

To further investigate the effects of 25 nM dasatinib, we conducted additional co-culture experiments using CAR19-NK-92 cells and CD19-positive tumor cell lines (Nalm-6 and Raji) at an effector-to-target ratio of 2:1 (**Figure 6**). CAR19-NK-92 cells were co-cultured under three conditions: in the absence of dasatinib (DMSO control), in the presence of dasatinib (25 nM), or following a 24-hour dasatinib treatment, the inhibitor was removed by washing before co-culturing with CD19-positive tumor cell lines. Under condition involving a 24-hour dasatinib treatment followed by washing, cytotoxicity was enhanced across all CAR19-NK-92 constructs in the presence of Raji cells (**Figure 6A**). In contrast, dasatinib treatment suppressed cytotoxicity across all CAR19-NK-92 constructs and non-CAR-NK-92 during active exposure (**Figures 6A, E**).

We also assessed CD107a expression and IFN-γ production by using CAR19-NK-92 cell effector function markers. In Raji cells, pretreatment with dasatinib significantly enhanced cytotoxicity, degranulation, and IFN-γ secretion across all CAR constructs as well as in non-transduced NK-92 cells, demonstrating an overall improvement in CAR19-NK-92 cell activity (**Figures 6B-D**). Interestingly, in the co-culture with Nalm-6 cells, only CAR19-NK-92 cells incorporating the 2B4 co-stimulatory molecule demonstrated increased cytotoxic activity (**Figure 6E**). In contrast to observed in the co-culture against Raji cells, in the assays with Nalm-6 cells, the enhancement of effector functions following dasatinib removal was observed exclusively in CAR19-NK-92 cells with 2B4ζ and 2B4D12 constructs, where were higher the percentage and intensity of CD107a positive cells as well as the IFN-γ production during co-culture (**Figures 6F-H**).

These results indicate that the presence of the 2B4 co-stimulatory domain amplifies the modulatory effects of dasatinib on CAR19-NK-92 cell cytotoxicity. Importantly, the selective enhancement observed against Nalm-6 cells suggests that CAR-dependent signaling is crucial for this effect, whereas the broader increase in activity against Raji cells may also involve modulation of endogenous NK cell receptors. Together, these findings highlight that dasatinib can differentially potentiate CAR-NK cell effector functions depending on both the co-stimulatory domain incorporated and the target cell ligand repertoire.

Next, we investigated whether transient dasatinib treatment could prevent exhaustion using a rechallenge assay. In this assay, the cytotoxic function of CAR19-NK-92 cells was evaluated *in vitro* over three days, with additional tumor targets introduced into the co-culture daily. CAR19-NK-92 cells were pre-treated with or without 25 nM dasatinib and co-cultured with Raji or Nalm-6 tumor cells at an effector-to-target (E:T) ratio of 1:5. The number of viable target cells was determined daily.

**Figure 7:**
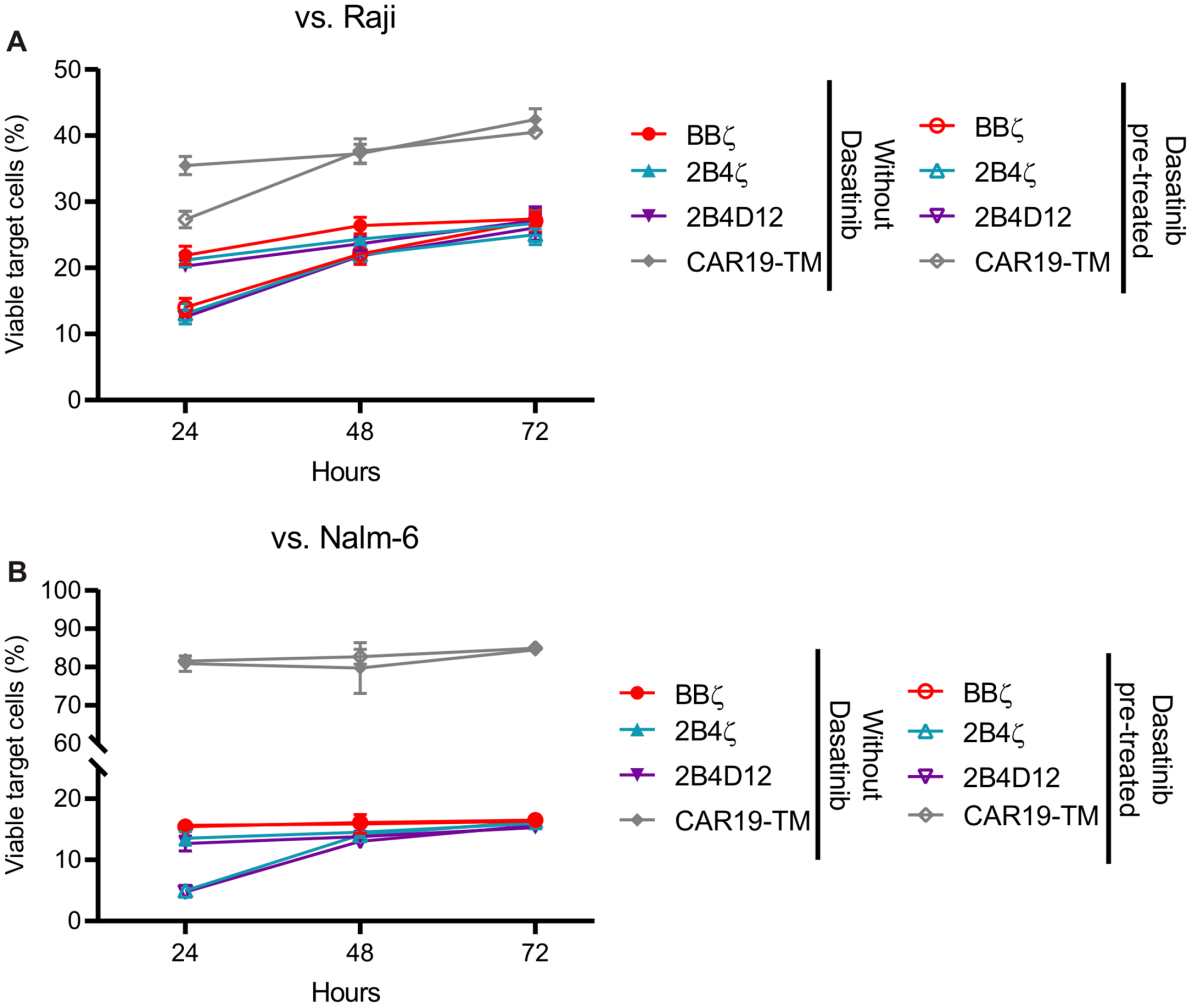
Evaluation of Dasatinib cytotoxicity enhancement upon rechallenge with target cells. CAR19-NK-92 cells were treated once with 25 nM or DMSO as a control for 24 hours, washed, and co-cultured with CD19 positive cell lines Raji or Nalm-6 at a 1:5 E:T ratio. Every 24 hours for 3 days, the remaining target cells were assessed for cell viability in flow cytometry, and fresh targets were added for co-culture, respecting the E:T ratio. **(A, B)** Scatter plot showing remaining viable Raji and Nalm-6, respectively, cells in co-culture with CAR19-NK-92 pre-treated or not with dasatinib at indicated time points.

During the first 24 hours, dasatinib pre-treated CAR19-NK-92 cells demonstrated enhanced cytotoxicity against Raji cells (**Figure 7A**) compared with untreated cells. However, after the first and second round of rechallenges, no significant differences in the target cell viability were observed between the dasatinib-treated and untreated groups (**Figure 7A**). In co-cultures with Nalm-6 cells, CAR constructs containing only 2B4ζ and 2B4D12 co-stimulatory domains reduced the number of viable target cells during the first 24 hours, indicating enhanced cytotoxicity (**Figure 7B**). Similar to the Raji co-culture, this effect diminished with subsequent rechallenges, and the cytotoxicity levels were comparable between the dasatinib-treated and untreated groups (**Figure 7B**). These findings suggest that although dasatinib initially enhances CAR19-NK-92 cell cytotoxicity, its effects are transient and do not prevent functional loss of cytotoxicity during prolonged or repeated tumor challenges.

### 3.6 2B4 cells pre-treated with dasatinib exhibit superior tumor control *in vivo*

**Figure 8:**
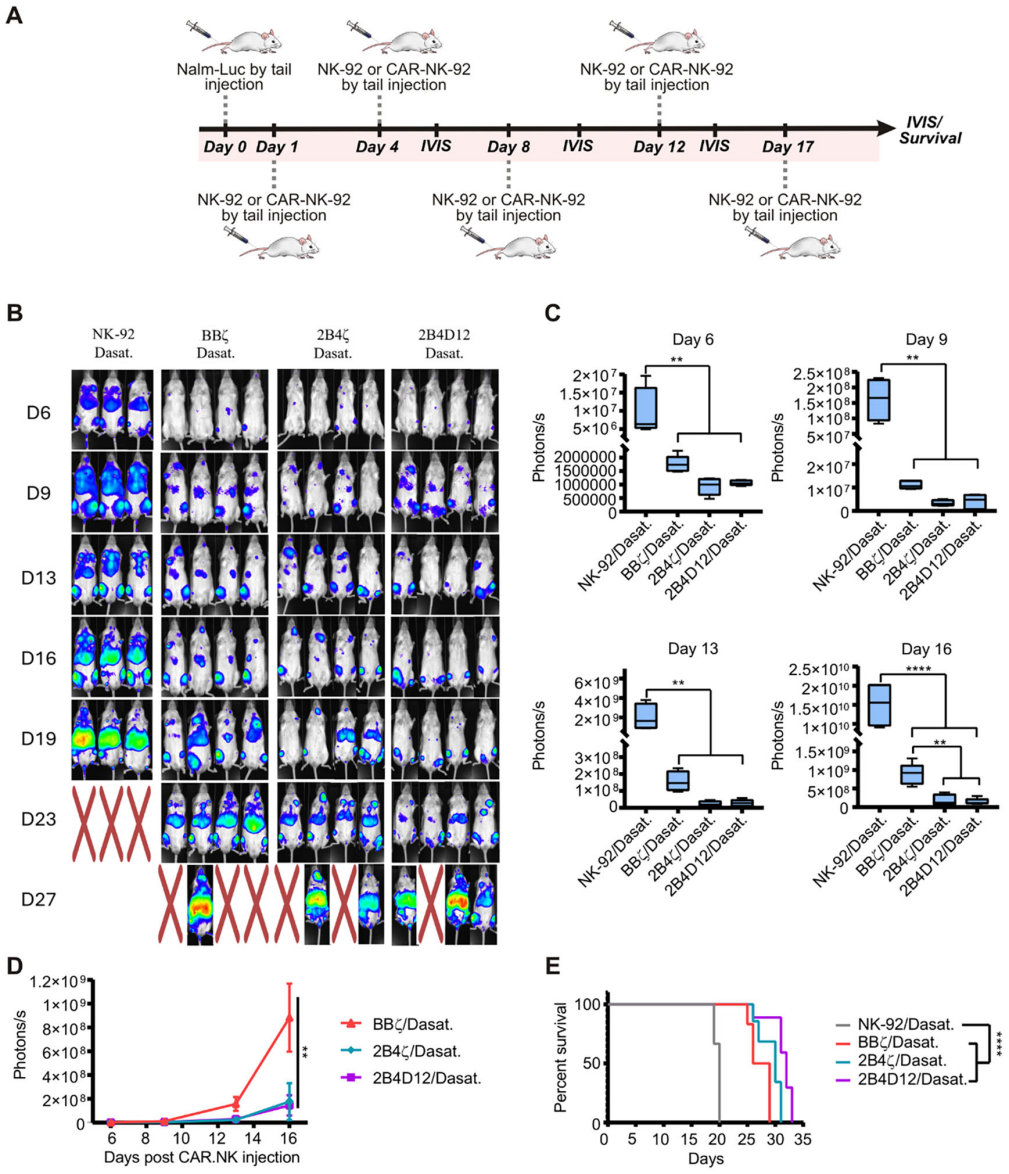
*In vivo* assessment of anti-tumor activity of CAR19-NK-92 cells following short-term Dasatinib treatment. **(A)** Experimental design. Mice were intravenously inoculated with 1×10^4^ Nalm-Luciferase cells on day 0 and treated with NK-92 or CAR19-NK-92 cells (BBζ, 2B4ζ, or 2B4D12). **(B)** Tumor progression was monitored by bioluminescence, with representative bioluminescence images shown. Mice marked with “X” indicate death during the experiment. **(C)** Comparison of the quantification of tumor burden from BLI signals in each treatment group on a specific day. Data are presented as box plots. Results are presented as mean ± SD, with significant differences determined by one-way ANOVA (****p < 0.001, ***p < 0.01, **p < 0.1). **(D)** Bioluminescence quantification showing tumor burden over time. Results are presented as mean ± SD, with significant differences determined by one-way ANOVA (**p < 0.1). **(E)** Survival analysis of the animals represented by the Kaplan-Meier curve. Statistically significant differences were determined by the Log-rank test (****p < 0.001).

We next evaluated the antitumor activity of dasatinib-pretreated CAR19-NK-92 cells *in vivo* using an NSG mouse model inoculated with Nalm-Luc cells (**Figure 8A**). Consistent with the *in vitro* findings, pretreatment with dasatinib selectively enhanced the efficacy of CAR constructs containing the 2B4 co-stimulatory domain. While non-transduced NK-92 cells failed to control tumor growth regardless of dasatinib exposure, and BBζ cells showed no significant improvement, both 2B4ζ and 2B4D12 cells exhibited superior tumor control following dasatinib pretreatment (**Figures 8B, C**). This effect was particularly evident on day 16, where bioluminescence signals were reduced compared with BBζ and non-transduced NK-92 groups (**Figures 8C, D**). Importantly, mice pretreated with dasatinib 2B4D12 cells displayed prolonged survival compared with all other groups (**Figure 8E**). These results demonstrate that dasatinib pretreatment recapitulates the *in vitro* phenotype, where enhanced activity was restricted to 2B4-based CARs, considering as target Nalm-6 cells, and translate this effect into improved resistance to *in vivo* tumor progression.

Overall, these results suggest that combining CAR.19-NK-92 cells with dasatinib may provide added benefit, particularly for constructs incorporating the 2B4 costimulatory domain. Alternatively, 2B4 domains may provide complementary signals that enhance degranulation and cytokine production following drug withdrawal, thereby amplifying antitumor activity. The enhanced efficacy observed in 2B4ζ and 2B4D12 cells possibly reflects the critical role of 2B4-dependent signaling pathways in driving antitumor responses and underscores the importance of selecting optimal co-stimulatory domains to maximize therapeutic outcomes.

## 4 Discussion

Currently, several CAR-T cell therapies have been approved and are available in the market, which have shown significant success in treating hematologic malignancies, such as leukemia and lymphoma (30–32). Despite the promising advancements in CAR-NK cell therapy (15), there are no approved CAR-NK products available. CAR-NK cells have shown potential advantages over CAR-T cells, including a lower risk of cytokine release syndrome and graft-versus-host disease, as well as the ability to target a broader range of tumors (15). However, several challenges must be addressed before CAR-NK cell therapy can become a viable clinical product. One challenge lies in the design of the CAR molecule itself, which directly affects efficacy, persistence, and functionality. Unlike CAR-T cells, NK cells use distinct activation pathways, and conventional co-stimulatory domains, such as 4-1BB and CD28 (which are optimized for T cells), may not provide optimal signaling for NK cell activation and persistence (33).

In CAR-T cell research, the co-stimulatory domain is a key factor that significantly influences the therapeutic success of CAR-T cells (34). To date, most engineered CAR-NK cells have adopted co-stimulatory domains derived from T cell-associated factors. Many clinical studies have focused on designing second-generation CARs with the CD28 domain (ClinicalTrials.gov Identifier: NCT03056339, NCT03579927), while others have used the 4-1BB domain (ClinicalTrials.gov Identifier: NCT02944162, NCT01974479, and NCT03941457). Additionally, third-generation CARs that combine both CD28 and 4-1BB domains or the addition of soluble cytokines such as IL-27 or complexes such as the IL-15/IL15Rα have been developed, showing potential for enhanced effectiveness when using a combination of these co-stimulatory signals (15, 27, 35). These efforts underline the critical role of co-stimulatory/intracellular domains selection in optimizing CAR NK cell function and improving anti-tumor responses.

Our study highlights two key strategies to optimize CAR-NK therapy: incorporating NK-specific co-stimulatory domains (2B4D12) and transient pharmacologic modulation with dasatinib. The use of 2B4D12 enhanced NK cytotoxic programming, as confirmed by transcriptomic and functional analyses. Additionally, dasatinib pretreatment provided a reversible mechanism where the observed suppression of cell activation, which demonstrated to suppress tonic signaling in CAR-T cells (25), augmented antitumor activity upon withdrawal.

Our *in vitro* results demonstrated that all CAR constructs effectively targeted the CD19-positive cells. Notably, CAR constructs incorporating the 2B4 co-stimulatory domain, in combination with either CD3ζ or DAP12, exhibited cytotoxic activity comparable to that of CAR19-BBζ. To better understand the potential pathway differences between 4-1BB and 2B4, we performed RNA-seq analysis, which revealed the upregulation of key effector molecules associated with cytotoxic functions in 2B4ζ and 2B4D12 CAR19-NK-92 cells. This suggests an enhanced capacity for degranulation and targeted cell lysis. A direct comparison between 2B4ζ and 2B4D12 indicated that 2B4D12 may further amplify NK activation pathways. Despite the transcriptional upregulation of cytotoxicity- and cytokine-related genes observed in the 2B4ζ and 2B4D12 constructs, these changes did not translate into significant differences in our *in vitro* assays. These discrepancies may also be influenced by NK-92–specific features. Unlike primary NK cells, NK-92 cells are of malignant origin, require continuous IL-2 supplementation, lack most functional KIRs and other regulatory receptors, and display a pre-activated cytotoxic phenotype with high basal levels of perforin and granzymes. Together, these characteristics may uncouple transcriptional changes from functional outcomes and highlight the limitations of this model in fully recapitulating the regulatory complexity of primary NK cells.

2B4 (CD244) is a SLAM-related receptor containing four immune-receptor tyrosine-based switch motifs (ITSMs) that regulate immune cell activity. Its ligand, CD48, is a GPI-anchored Ig-like protein expressed in most hematologic cells, including NK cells. Upon interaction with CD48, 2B4 undergoes ITSM phosphorylation, leading to the recruitment of SLAM-associated protein (SAP) and EWS-Fli1-activated transcript 2 (EAT-2) (36). SAP facilitates the recruitment of Fyn kinase, which phosphorylates phospholipase C-γ (PLC-γ) or Vav-1, activates ERK signaling, enhances NK cell cytotoxicity, and promotes the secretion of the pro-inflammatory cytokines IFN-γ and TNF-α. EAT-2 further links 2B4 to PLC-γ and ERK, strengthening NK cell activation and accelerating the release of cytotoxic granules (36).

Similarly, DAP12 (TYROBP) is an adaptor protein involved in immune cell activation that contains an immunoreceptor tyrosine-based activation motif (ITAM) in its intracellular domain. It interacts with various activating receptors on natural killer (NK) cells, macrophages, and other myeloid cells. Upon ligand binding, DAP12 is phosphorylated by Src family kinases, leading to recruitment of Syk or ZAP-70. This cascade activates downstream pathways, including PLC-γ, mitogen-activated protein kinase (MAPK), and nuclear factor-κB (NF-κB), ultimately promoting cytotoxicity, cytokine production (e.g., IFN-γ and TNF-α), and immune cell activation (37).

Several prior studies have investigated how choice of intracellular costimulatory motifs shapes CAR-NK function. Incorporation of CD28 into CARs enhances recruitment of kinases such as LCK and ZAP70 and can augment proximal CAR signaling in primary NK cells, improving effector responses in preclinical models (38). Previous studies have shown that CAR-NK cells using the 2B4 intracellular domain as a co-stimulatory module promote the phosphorylation of PLC-γ, Vav-1, and ERK, reinforcing NK cell activation and NK-specific signaling motifs such as 2B4, DAP10 and DAP12 have likewise been shown to modulate NK activation and to improve cytotoxicity in multiple CAR-NK systems (16, 17, 39, 40) These findings suggest that the 2B4 intracellular domain may serve as a more effective co-stimulatory domain in CAR-NK cells compared to 4-1BB (17). Our findings align with these observations, showing that 2B4-based architectures (2B4-CD3ζ and 2B4-DAP12) enhance NK-92 cytotoxic activity and enrich transcriptional pathways associated with effector function when compared to 4-1BB-CD3ζ, suggesting that NK-centric costimulatory design can yield distinct functional advantages. Additional comparative analyses including a broader range of CAR architectures will be important to further delineate how specific costimulatory and signaling domain combinations influence NK cell activation and function.

Dasatinib is a broad-spectrum TKI that targets multiple kinases including Src family kinases (SFKs), LCK, Fyn, ZAP-70, and Syk. In the context of CAR-T cells therapy, it was demonstrated that it can act as a swift-off inducer, temporarily inhibiting cell activation (25). Accordingly, it was demonstrated that it also transiently inhibits activation signaling in NK-92 cells, however, upon drug removal, it could potentially lead to a heightened activation response when co-culture against tumor target cells, as the inhibited pathways are reactivated (41, 42). Based on these reports, we sought to investigate the effects of dasatinib on CAR-engineered NK-92 cells.

Our findings demonstrated that transient dasatinib treatment enhanced CAR19-NK-92 cytotoxicity upon drug withdrawal, particularly in constructs incorporating the 2B4 co-stimulatory domain. This enhancement is driven by increased degranulation and IFN-γ secretion, with effects varying according to the target cell type. Compared with Nalm-6 cells, improved cytotoxicity appears to depend primarily on CAR signaling, whereas in Raji cells, dasatinib may also influence additional NK cell receptors, further enhancing NK cell activity. It was demonstrated that the enhancing cell activation upon drug withdrawal is associated mainly by the phosphorylation intensity augmented in the Vav pathway, that is directly linked to the 2B4 stimulatory domains (41), indicating that the differential response may be associated to the CAR co-stimulatory domain.

These effects were also observed *in vivo*. Although CAR19-BBζ cells showed no significant benefit, CAR19-2B4ζ and CAR19-2B4D12 constructs exhibited superior tumor control, with CAR19-2B4D12 demonstrating the most pronounced effect. The significant reduction in tumor burden and prolonged survival in mice treated with CAR19-2B4D12 plus dasatinib suggests that transient kinase inhibition may enhance CAR19-NK-92 efficacy. Collectively, these findings support the further investigation of dasatinib as a tool to optimize CAR-NK cell-based immunotherapies.

It is important to acknowledge that the dasatinib experiments reported in this study were conducted using the NK-92 cell line, and the effects observed may not fully translate to primary human NK cells. NK-92 displays constitutive activation of downstream signaling pathways, which may influence its sensitivity to tyrosine kinase inhibition, whereas primary NK cells are heterogeneous with diverse activation thresholds and inhibitory receptor repertoires, factors that could lead to differential responses to dasatinib exposure and withdrawal. Indeed, previous reports have shown that dasatinib can variably affect NK cell function depending on cellular context and drug regime treatment (42–44).

The NK-92 platform was selected as a discovery tool to compare CAR designs and to explore pharmacological modulation in a reproducible setting, rather than as a direct therapeutic product. Although NK-92 cells used for clinical translation require irradiation and lack persistence (45, 46), they remain a valuable model for elucidating signaling mechanisms and for the rapid screening of CAR configurations. The demonstration that 2B4-based signaling domains and transient dasatinib exposure enhance NK effector functions provides design principles that can be applied to primary NK cells, where persistence, *in vivo* expansion, and long-term function are critical.

Because IL-15 has been shown to independently enhance NK-92 effector functions (27) our current design does not allow us to fully disentangle the contribution of IL-15 secretion from CAR-mediated activity. This limitation may therefore underestimate the baseline functional capacity of IL-15–engineered NK-92 cells and complicates direct comparisons with CAR-IL-15 constructs.

In conclusion, our study highlights the potential of transient dasatinib treatment as a strategy for enhancing CAR-NK cell function. By temporarily inhibiting key kinases, dasatinib treatment was able to lead the cells to a more robust cytotoxic response after drug removal. This effect was particularly evident in CAR19-NK-92 cells incorporating the 2B4 co-stimulatory domain in combination with the cell line Nalm-6, which exhibited increased degranulation, IFN-γ secretion, and increased tumor cell death both *in vitro* and *in vivo*. Notably, CAR19-2B4D12 cells demonstrated the greatest benefit, achieving superior tumor control and prolonged survival in an NSG mouse model. These findings suggest that combining CAR with associated NK co-stimulatory molecules and transient dasatinib treatment could optimize CAR-NK therapy, ensuring greater efficacy.

## Data Availability

RNA-seq data and supporting datasets are available at the NCBI Gene Expression Omnibus (GEO) and are accessible through GEO Series accession number GSE306760.

## Supplementary material

Supplementary Figure 1 CD19 expression in different cell lines. Flow cytometry analysis of CD19 expression in Nalm-6, Namalwa, Raji, and K562 cell lines. Cells were stained with an anti-CD19 antibody (red histograms) or an isotype control (gray histograms, ISO) and analyzed for surface CD19 expression. Nalm-6, Namalwa, and Raji cells exhibit CD19 positivity, while K562 cells serve as a CD19-negative control.

Supplementary Figure 2 Evaluation of cytotoxicity of NK-92 and CAR19-NK-92 cells treated with dasatinib against CD19-positive tumor cells. **(A)** Representative illustration of the experimental procedure for treating NK-92 cells with dasatinib and co-culturing them with target cells. **(B and C)** NK-92 and CAR19-NK-92 cells were treated for 24 hours with different concentrations of dasatinib (0, 10, 25, 50, 100, and 200 nM) or DMSO (control). After treatment, cells were washed with PBS and co-cultured for 5 hours with PKH67-labeled target cells at an effector-to-target (E:T) ratio of 2:1. Target cell viability was determined using flow cytometry. Heatmaps show the mean cytotoxicity (%), with standard deviations indicated in parentheses. n = 3. Illustration created using https://www.BioRender.com.
