# Supplementary figures and images for: 2B4 co-stimulation and dasatinib modulation enhance anti-CD19 CAR-NK-92 cell cytotoxicity
Source: Front Immunol. 2025 Dec 12;16:1675877. doi: 10.3389/fimmu.2025.1675877 (PMC12741080; doi:10.3389/fimmu.2025.1675877)

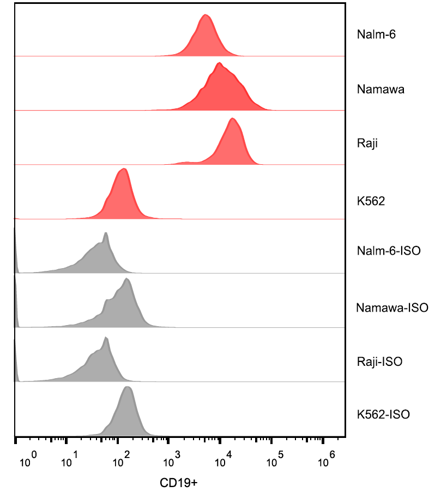

Supplement: Supplementary Figure 1 — CD19 expression in different cell lines. Flow cytometry analysis of CD19 expression in Nalm-6, Namalwa, Raji, and K562 cell lines. Cells were stained with an anti-CD19 antibody (red histograms) or an isotype control (gray histograms, ISO) and analyzed for surface CD19 expression. Nalm-6, Namalwa, and Raji cells exhibit CD19 positivity, while K562 cells serve as a CD19-negative control. [file Image1.tiff]

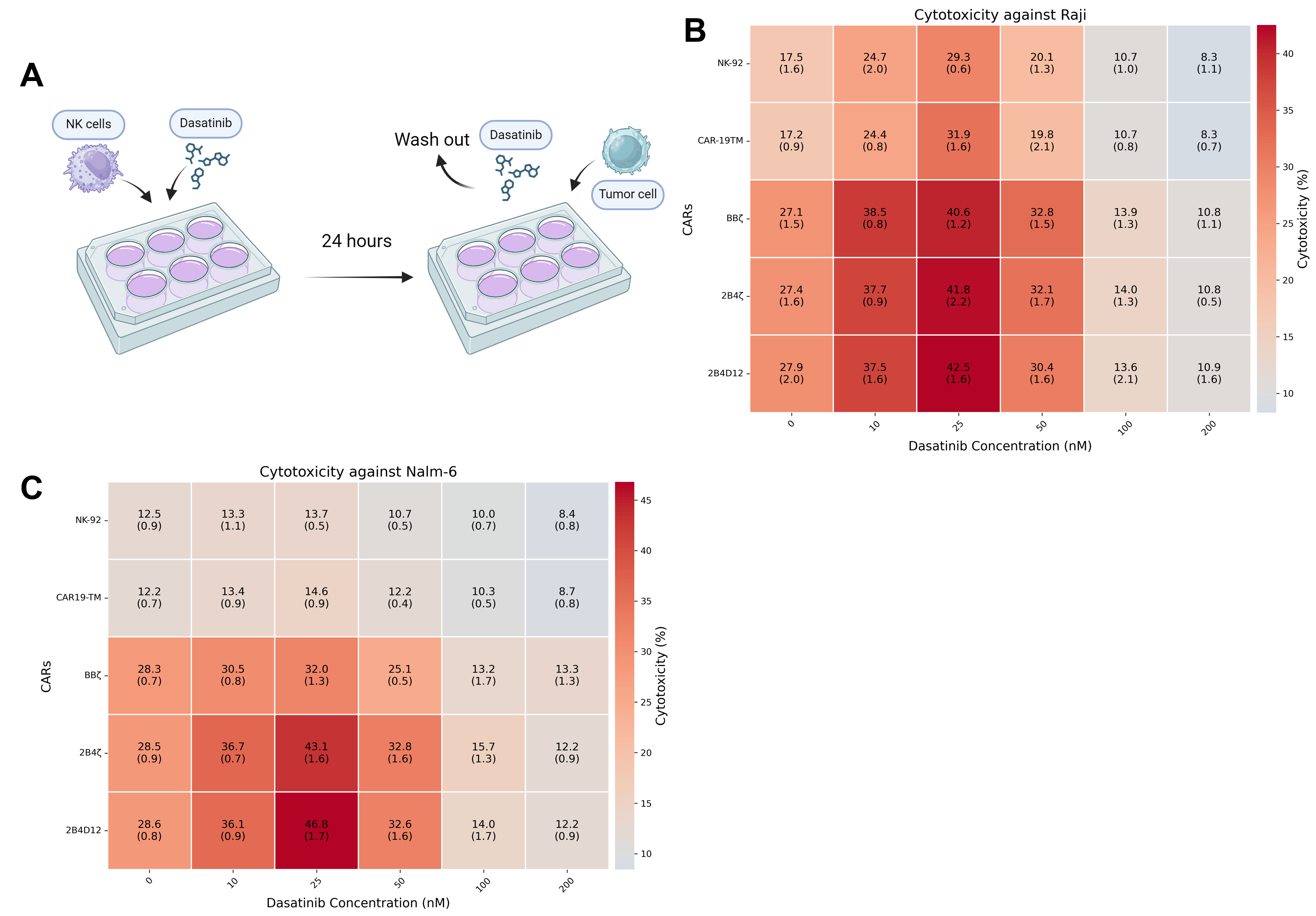

Supplement: Supplementary Figure 2 — Evaluation of cytotoxicity of NK-92 and CAR19-NK-92 cells treated with dasatinib against CD19-positive tumor cells. (A) Representative illustration of the experimental procedure for treating NK-92 cells with dasatinib and co-culturing them with target cells. (B and C) NK-92 and CAR19-NK-92 cells were treated for 24 hours with different concentrations of dasatinib (0, 10, 25, 50, 100, and 200 nM) or DMSO (control). After treatment, cells were washed with PBS and co-cultured for 5 hours with PKH67-labeled target cells at an effector-to-target (E:T) ratio of 2:1. Target cell viability was determined using flow cytometry. Heatmaps show the mean cytotoxicity (%), with standard deviations indicated in parentheses. n = 3. Illustration created using https://www.BioRender.com. [file Image2.tiff]
